# Recent and Future Advances in the Chemoenzymatic Synthesis of Homogeneous Glycans for Bacterial Glycoconjugate Vaccine Development

**DOI:** 10.3390/vaccines9091021

**Published:** 2021-09-14

**Authors:** Ayobami Adegbite, Pumtiwitt C. McCarthy

**Affiliations:** 1 Bioenvironmental Sciences Program, Morgan State University, Baltimore, MD 21251, USA; ayade9@morgan.edu; 2 Department of Chemistry, Morgan State University, Baltimore, MD 21251, USA

**Keywords:** glycoconjugate vaccines, bacterial pathogens, chemoenzymatic synthesis

## Abstract

Vaccines are important in preventing disease outbreaks and controlling the spread of disease in a population. A variety of vaccines exist, including subunit, recombinant, and conjugate vaccines. Glycoconjugate vaccines have been an important tool to fight against diseases caused by a number of bacteria. Glycoconjugate vaccines are often heterogeneous. Vaccines of the future are becoming more rationally designed to have a defined oligosaccharide chain length and position of conjugation. Homogenous vaccines could play an important role in assessing the relationship between vaccine structure and immune response. This review focuses on recent advances in the chemoenzymatic production of defined bacterial oligosaccharides for vaccine development with a focus on *Neisseria meningitidis* and selected WHO-prioritized antibacterial resistant-pathogens. We also provide some perspective on future advances in the chemoenzymatic synthesis of well-defined oligosaccharides.

## 1. Introduction

Vaccines are preventative measures that aid in controlling the spread of disease in a population [1,2]. For example, this is the second year of the global pandemic caused by SARS-CoV2, and the distribution of vaccines to a critical mass of the population will be crucial to slowing the continuing spread of the coronavirus and decreasing the mortality caused by the disease [3]. Vaccines work by inducing short-lived or long-lasting immune response in humans and animals [4,5]. Vaccines contain antigens that elicit the formation of antibodies toward a particular pathogen through an adaptive immune response [6]. Many types of vaccines exist, including live-attenuated vaccines, inactivated vaccines, subunit, recombinant, and conjugate vaccines [2]. Live-attenuated and inactivated vaccines contain components from the natural pathogen (such as outer membrane vesicles) that can act as self-adjuvants [7]. Adjuvants are non-immunogenic compounds that act to increase the immune response by activating antigen-presenting cells. Other types of vaccines (primarily subunit and recombinant) require the use of adjuvants [7,8]. Glycolipids are one class of adjuvant that has been used. For example, QS-21, an amphipathic saponin has been used as an adjuvant to induce T-cell dependent immune responses in veterinary and human vaccines [9]. Similarly, mimics of phosphatidylinositol mannosides (PIMs) have been studied for their ability to bind to DC-SIGN on dendritic cells [10,11].

Glycoconjugate vaccines have been one tool used to fight against diseases caused by a number of bacteria. Carbohydrates (monosaccharides, oligosaccharides, and polysaccharides) play important functional roles in bacteria. Glycoconjugate vaccines contain oligosaccharides that are attached to a carrier protein. The covalent linkage of polysaccharide to a carrier protein transforms the immune response from a T-cell-independent to a T-cell-dependent response, leading to the production of IgG-based antibody responses [5]. These sugar fragments can be directly isolated from the pathogen or obtained through synthesis. One of the earliest example of a successful glycoconjugate vaccine using pathogen-derived oligosaccharides was *Haemophilus influenzae type b* [12]. To make this type of vaccine, polysaccharides are obtained from the organism, acid-hydrolyzed to oligosaccharides, and derivatized before conjugation to a derivatized carrier protein [13]. The resulting conjugates are often heterogeneous with random multiple sites of attachment of sugars to protein [14]. Despite this fact, these vaccines are successful in protecting individuals from several different pathogens. Vaccines of the future could be rationally designed to have a defined oligosaccharide chain length and position of conjugation. These types of vaccines will play important roles in defining the relationship between vaccine structure and the strength of the immune response generated.

Chemoenzymatic synthesis is a promising route to obtaining well-defined homogeneous vaccines. Chemical synthesis of oligosaccharides has also been a successful strategy for medically relevant oligosaccharides [15]. In comparison to only chemical synthesis, chemoenzymatic synthesis offers some advantages, such as milder reaction conditions and no need for carbohydrate-protecting groups because of regiospecificity (Table 1). Typically, chemically synthesized substrates are reacted with carbohydrate-producing enzymes under controlled conditions (with a balance of temperature, pH, enzyme concentration, metal ion concentration, acceptor substrate, donor sugar concentration) to produce the desired oligosaccharides [16]. Enzymatic steps could be carried out in tandem or at the same time. One-pot multienzyme synthesis (OPME) approaches involve the combination of many steps in one reaction vessel [16]. Besides the specific enzyme present within the system, recycling enzymes that convert byproducts (free nucleotides) into substrates (nucleotide donor sugars) could also be present to drive efficient oligosaccharide production. This review focuses on recent advances in the chemoenzymatic production of defined bacterial oligosaccharides with a focus on *Neisseria meningitidis* and selected WHO-prioritized antibacterial-resistant pathogens. We also provide some perspective on future advances in the chemoenzymatic synthesis of well-defined oligosaccharides.

## 2. Chemoenzymatic Synthesis of Meningococcal Oligosaccharides

**Figure 1:**
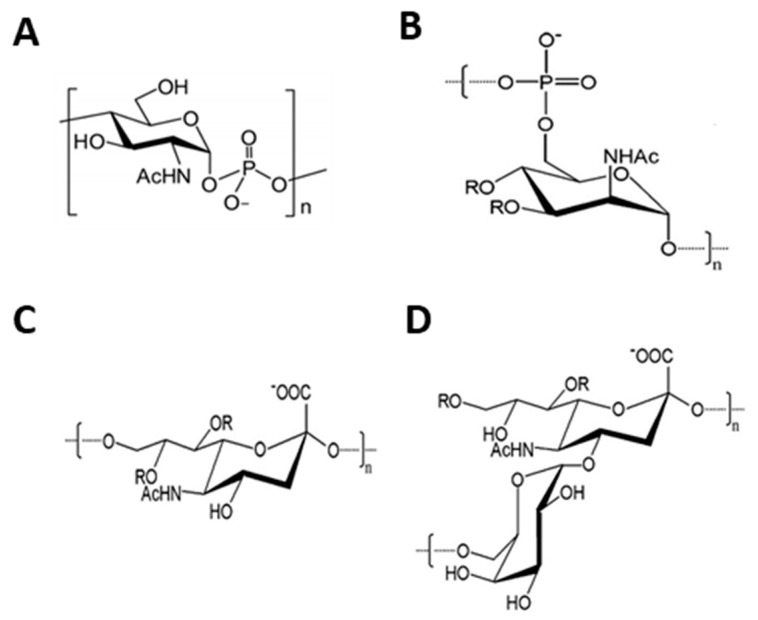
Monosaccharide units of *N. meningitidis* serogroup polysaccharides discussed in this review. (**A**) serogroup X; (**B**) serogroup A; (**C**) serogroup C; (**D**) serogroup W.

*N. meningitidis* is a Gram-negative bacterium and one of the leading causes of bacterial meningitis worldwide. Of the 13 identified serogroups, six have been identified to be pathogenic. The six pathogenic ones are A, B, C, X, W, and Y [17,18,19]. Glycoconjugate vaccines exist for serogroups A, C, W, and Y. A protein-based vaccine is available for serogroup B, and there is no licensed vaccine of either type for serogroup X. There has notably been quite a bit of development in the chemoenzymatic synthesis of meningococcal oligosaccharides (Figure 1), as the enzymes responsible for synthesis in the various serogroups have been characterized by different research groups [20,21,22,23,24,25,26,27,28,29,30].

### 2.1. N. meningitidis Serogroups A and X

In studies conducted by Fiebig et al., researchers characterized the capsule polymerases from *N. meningitidis* serogroup A (CsaB) and serogroup X (CsxA) [28,31]. These enzymes are both members of the *stealth* protein family; CsaB synthesizes a homopolymer of α1→6 N-acetyl-mannosamine phosphate, and CsxA synthesizes a homopolymer of α1→4N-acetylglucosamine phosphate. The heteropolymer from serogroup A also has variable degrees of O-acetylation at carbon 3 and carbon 4. In previous work, CsaB was confirmed as the transferase within the capsule gene cluster for serogroup A [28]. In this work, the CsaB and CsxA were characterized in terms of whether product formation was processive or distributive. Recombinant CsaB exhibited distributive product formation. Recombinant CsxA was converted from a processive to a distributive enzyme through the removal of 98 amino acids from the C-terminus in combination with the removal of 58 amino acids from the N-terminus. This shift in enzyme activity afforded control of the size of oligosaccharides generated. Oligosaccharides with an average degree of polymerization (DP) of 15 were obtained using this truncated protein immobilized on nickel resin with specific donor/acceptor ratios and a DP5 acceptor. This proof-of-concept work set the foundation for research using these methods for production of an immunogenic glyccoconjugate. Oldrini and colleagues used a one-pot multienzyme approach to synthesize serogroup X oligomers from a synthetic trisaccharide of the serogroup X polymer containing a propyl-amine at the reducing end using the same setup for immobilized CsxA [32]. The enzymatically-produced oligomers with (average DP10) were conjugated directly to a CRM_197_ carrier protein using N-hydroxysuccinmidyl adipic acid chemistry. Three rounds of immunization were performed with these conjugates in comparison to CRM_197_ conjugates containing naturally-derived and enzymatically synthesized oligosaccharides respectively. Antibodies produced from immunization with both conjugates were reactive against serogroup X capsular poysaccharide. In addition, these antibodies were bactericidal in a serum bactericidal assay.

### 2.2. Neisseria meningitidis Serogroup C

McCarthy et al. used a chemoenzymatic approach to produce a well defined vaccine candidate using click chemistry conjugation [24,33]. A chemically synthesized azido-containing lactoside, 9-azidononanyllactoside, was used as a substrate for *Camplyobacter jejuni* CSTII sialyltransferase and the *N. meningitidis* serogroup C polysialytransferase. The inhibitor CMP-9-deoxy-NeuNAc was used to control the chain length of the oligosialic acids produced yielding polymers with median DP25 and DP51, respectively. These oligosialic acids were conjugated to alkyne-modified receptor fragment of the tetanus toxin (TetHc) protein as a carrier. After injection with the glycoconjugates, mice sera showed higher IgG titers over control mice that received unconjugated sialic acids. Antibodies from immunized mice were reactive against serogroup C polysaccharide.

### 2.3. Neisseria meningitidis Serogroup W

The *N. meningitidis* serogroup W capsule polymerase synthesizes a heteropolymer of sialic acid α(2,6)-linked to galactose by glycosidic linkage [21,22,30]. This polysaccharide is *O*-acetylated at positions C7 and C9 [34]. Li et al. synthesized structurally defined synthetic serogroup W oligosaccharides tagged with a hydrophobic chromophore (2-O-(N-benzyloxycarbonyl)aminopropyl α-N-acetylneuraminide) using the capsule polymerase, *N. meningitidis* CMP-sialic acid synthetase, *Streptococcus pneumoniae* galactokinase, *Bifidobacterium longum* UDP-sugar pyrophosphorylase and *Pasteurella multocida* inorganic pyrophosphatase in a sequential one-pot multienzyme platform [27]. Using this method, defined oligomers of DP > 65 were produced. Overall, the work showed that synthesis of size-controlled oligosaccharides can be produced effectively through modulation of the donor:acceptor ratio. These methods are promising for the development of conjugatable oligosaccharides for vaccine development.

*N. meningitidis* is one type of bacteria for which there are licensed vaccines on the market. In addition, there is not a large threat of antibiotic resistance with this pathogen. Quite recently, however, there have been reports of an uptick in penicillin- and/or ciprofloxacin-resistant cases *N. meningitidis* serogroup Y [35]. There are several bacterial species that are resistant to antibiotics. In the next section, we discuss vaccination as a strategy to prevent antibacterial resistance and describe chemoenzymatic synthetic efforts of some oligosaccharides from selected bacterial pathogens.

## 3. Enzymatic Synthesis of Oligosaccharides from Antibiotic-Resistant Bacteria

Antibiotic resistance is a global problem [36]. Bacteria are continually evolving to incorporate mechanisms that weaken the effect of antibiotics. The World Health Organization (WHO) has developed a Global Action Plan to combat antimicrobial resistance [37] The five strategic goals of this plan are: “(1) to improve awareness and understanding of antimicrobial resistance; (2) to strengthen knowledge through surveillance and research; (3) to reduce the incidence of infection; (4) to optimize the use of antimicrobial agents; and (5) to develop the economic case for sustainable investment that takes account of the needs of all countries, and increase investment in new medicines, diagnostic tools, vaccines and other interventions”. Vaccine development is a proactive method to help lower antibiotic resistance. By producing vaccines against antibiotic-resistant pathogens, we can provide crucial protection to a population before they are sickened by disease. The WHO has four levels of priority for pathogens that they are targeting for research and development to prevent antibiotic resistance. These levels are highest priority, critical priority, high priority, and medium priority (Table 2).

There have been many efforts towards the chemical synthesis of oligosaccharides for homogeneous vaccine development against priority pathogens including *Staphylococcus aureus*, *Streptococcus pneumoniae*, *Hemophilus influenzae b* and *Shigella* [38,39,40,41,42,43,44]. In this section, we describe some of the advances in the chemoenzymatic/enzymatic synthesis of oligosaccharides from select WHO priority pathogens.

### 3.1. Highest Priority: Mycobacterium tuberculosis

**Figure 2:**
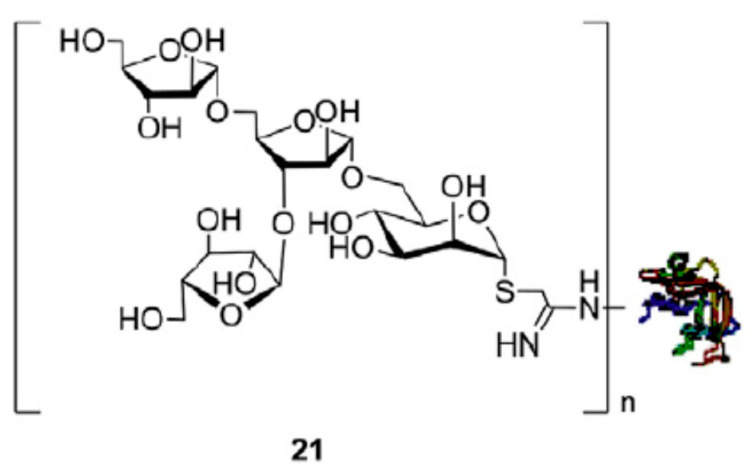
Glycoconjugate of ManAra3-rHSA. Reprinted from Li, Z.; Bavaro, T.; Tengattini, S.; Bernardini, R.; Mattei, M.; Annunziata, F.; Cole, R.B.; Zheng, C.; Sollogoub, M.; Tamborini, L., et al. Chemoenzymatic synthesis of arabinomannan (am) glycoconjugates as potential vaccines for tuberculosis. Eur J Med Chem 2020, 204, 11257 with permission from Elsevier [47].

Tuberculosis is caused by the pathogen *Mycobacterium tuberculosis*. The disease is one of the leading cause of infectious-disease-related death in the world [45]. The only current vaccine againt the pathogen is the BCG live-attenuated vaccine which is believed to be 50% effective and potentially has effectiveness for up to 20 years [46]. In recent work by Li and colleagues, a chemoenzymatic approach was used to facilitate the synthesis of oligosaccharides derived from lipoarabinomannan (LAM) for use in potential vaccine candidates [47]. Lipoarabinomannan and lipomannan are structural components of the *M. tuberculosis* cell wall and may contribute to antibiotic resistance [48]. Arabinomannan (AM) is what contributes to virulence in LAM and has internal α(1 → 6) and α(1 → 2) mannose covered by a branched arabinan domain. At the non-reducing end of this domain, a common motif of β(1 → 2) arabinose linked to α(1 → 5) and α(1 → 3) aribanose is found. Li et al. synthesized three oligosaccharides containing this motif (ManAra3, containing mannose linked to three arabinose sugars; Man3Ara3, containing three mannose linked to three arabinose sugars; and Man3Ara, containing three mannose linked to arabinose). In their work, a chemoenzymatic approach was developed using enzymatic hydrolysis by a resin-bound lipase B from *Candida antarctica* (CALB) as a step in synthetic flow chemistry [49,50,51]. This enzyme was able to site-selectively remove acetyl groups from a peracetylated arabinose thioglycoside, which was an important precursor in their synthetic scheme to obtain the ManAra oligosaccharides. The resulting oligosaccharides were conjugated to recombinant human serum albumin (rHSA), and ELISA tests were carried out using serum from patients infected with tuberculosis to determine whether the antibodies present could bind to the synthetic glycoconjugates. Results showed that the ManAra3-rHSA had a higher reaction compared to other glycoconjuates, which may indicate that this motif is a component of the human epitope (Figure 2).

### 3.2. Critical Priority: Klebsiella pneumoniae

**Figure 3:**
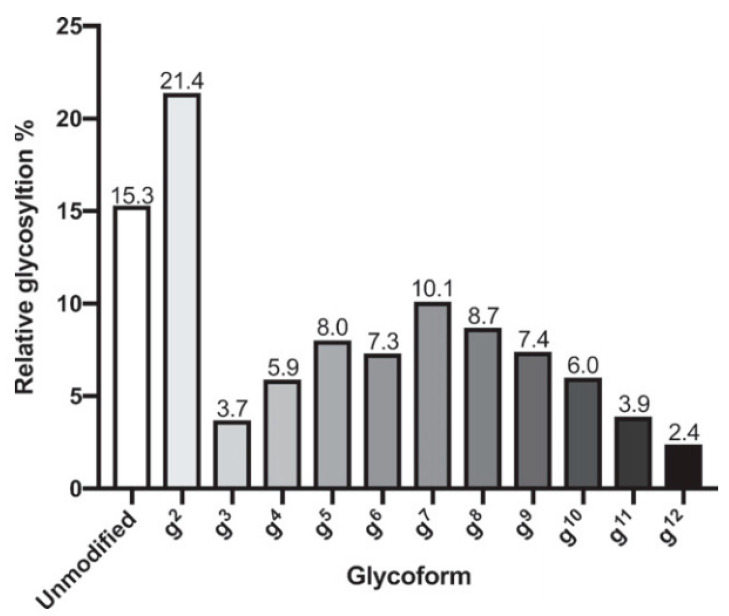
Quantification of the relative abundance of each K2 glycoform in K2-EPA. From Feldman et al. Ref. [53].

*K. pneumoniae* is a Gram-negative bacterium from the *Enterobacteriaceae* family. It is one type of nosocomial (hospital-acquired) infection, which usually affects immunocompromised individuals. Many (more than 80) serotypes of *K. pneumoniae* have been identified, and the most virulent serotypes are serotypes 1 and 2 (K1 and K2). These serotypes are responsible for the hypervirulent form of *K. pneumoniae* that are resistant to antibiotics and are transmitted through community-spread [52]. These serotypes contain capsular polysaccharides with glucose at the reducing end. Feldman et al. produced recombinant glycoconjugate vaccines against *Klebsiella pneumonia* serotypes 1 and 2 within *E. coli* cells using bioconjugation [53]. Glycoconjugate vaccines produced through bioconjugation use oligosaccharyltransferases to transfer polysaccharides from lipid carriers to a target carrier protein in the periplasm of Gram-negative bacteria [54]. In this work, researchers used PglS (from *Acinetobacter baylyi* ADP1), which has a broader substrate specificity and can transfer oligosaccharides that have glucose at the reducing end. Through glycoengineering of an *E. coli* strain (CLM37) to include the genes for capsular polysaccharide synthesis of *K. pneumoniae* strains K1 and K2 and the co-expression of a plasmid containing the transcriptional activator regulator of mucoid phenotype A (RmpA), the researchers successfully produced the K1 and K2 oligosaccharide antigens. Within their bioconjugation platform, the researchers also had an EPA-ComP fusion as a carrier protein—ComP is the acceptor for PglS, and EPA is the exotoxin A from *Pseudomonas aeruginosa*. Glycoconjugate formation was confirmed through characterization by NMR, immunoblotting, and mass spectrometry. For the K2-EPA, the most prevalent glycoform was two repeats of the core structure (g^2^) (Figure 3). The K1-EPA, K2-EPA, and K1/K2 (bivalent) glycoconjugates produced from these processes were evaluated for their immunogenicity in mice. Serum antibodies from immunized mice induced serotype-specific IgG responses from mice when compared to placebo. The bivalent glycoconjugates produced antibodies against both serotypes. In mice survival studies, the bivalent conjugate increased the survival of mice even at very high challenge of the K1 or K2 serotype dose. This work describes remarkable advances towards the development of a vaccine against hypervirulent *Klebsiella*. Bioconjugation has also been used for vaccine candidates against *Shigella flexneri* [55].

## 4. Future Directions

The use of chemoenzymatic/enzymatic methods in synthesizing oligosaccharides will continue to positively impact the production of homogeneous vaccines. Leveraging some of the recent advances in technologies described below will make this a reality.

### 4.1. Computational Methods: Molecular Dynamics Simulations of Antigens

Computational modeling and molecular dynamics (MD) simulations of carbohydrates can provide insight into how these structures may interact with binding partners and provide clarity on structural epitopes in concert with analytical characterization [56]. In recent work by Kuttel, Berti, and Ravenscroft, molecular modeling and MD was performed to investigate the role chain flexibility could have in the conjugation efficiency and valency in the production of monovalent glycoconjugate vaccines (containing one serogroup) against *N. meningitidis* serogroups C, W, and Y, which use CRM_197_ as a carrier [57]. The conjugates used in these studies were oligosaccharides of 18 repeating units (equal to 18 monosaccharides in the serogroup C homopolymer and equal to 36 monosaccharides for the heteropolymer). Kuttel et al. found that there were differences in flexibility in these oligosaccharides. In order of flexibility, the polymer with the most flexibility was NmW (NmW > NmY > NmC). More flexibility and size may contribute to the differences in the number of chains attached to CRM_197_. If oligosaccharides conjugated to the protein are flexible, this may lead to inaccessibility to some derivatization sites for coupling. It has been determined through previous experimentation that the NmW vaccine has the highest coupling ratio (6.9), followed by NmC (5.6), and, lastly, that NmY has the lowest (4.5). The work by Kuttel and colleagues has provided some potential insight into antigen flexibility that can be verified experimentally and can be used to inform the design of well-defined vaccines. In other work by Henriques and coworkers, molecular modeling and docking studies were complementary components of a multidisciplinary approach including saturation transfer difference NMR (STD-NMR), X-ray crystallography, and surface plasmon resonance to aid researchers in determining the role *O*-acetylation plays as a NmA structural epitope [58]. Docking studies with modeled oligosaccharides displaying varying O-acetylation patterns coupled with SPR studies revealed that the 3-*O*-acetylation was critical to the binding of the Fab region of the antibody.

Work by Zhang et al. described the use of STD-NMR spectroscopy and molecular dynamics simulations to determine the secondary structure of chemically synthesized *S. pneumoniae* serotype one polysaccharide (Sp1) oligosaccharide antigens that can mimic natural polysaccharides [59]. This pathogen is classified as medium priority by the WHO. There are currently licensed vaccines against various serotypes of *S. pneumonia* (i.e., Prevnar 13, which includes Sp1) yet the structure/immunogenicity ratio is still not entirely known. The repeating unit of the Sp1 polysaccharide contains negative and positive charge and contains a rare monosaccharide 2-acetamido-4-amino-2,4,6-trideoxy-D-galactose (D-AAT) linked to a disaccharide of galacturonic acid. Researchers sought to further understand the molecular mechanism of antigen binding to the elicited antibodies and how this binding is affected by oligosaccharide size. Work from this study used chemical synthesis to create defined oligomers of Sp1 (trimer, hexamer, nonamer, and dodecamer). MD simulations of these oligomers predicted a helical structure for the nonamer and dodecamer, and STD-NMR spectroscopy confirmed this. ELISA studies indicated that the nonasaccharide and dodecasaccharide had the binding effect that was closer to the binding of the natural polysaccharide then other oligosaccharides. The revelations from this study make it possible to determine the optimal antigen length for efficient binding to antibodies for Sp1.

Molecular dynamics simulations aided in the determination of the dominant conformation of oligosaccharides in a glycoconjugate vaccine against invasive non-typhoidal *Salmonella* (NTS) with serovars-enteritidis (SE) and typhimurium (STm) [60]. The glycoconjugate vaccine candidate, consisting of STm core and O-polysaccharide (COPS) conjugated to phase 1 flagellin protein from the same serovar. Mice injected with STm blood isolate were immunized using the vaccine candidate and were found to be immunogenic. In their work, STm phase 1 flagellin proteins and COPS were purified and characterized and further used to immunize mice who were challenged with Stm blood isolates. The chemistry of conjugation, valency of COPS, the effects of carrier protein, and *O*-acetylation status were also investigated. To complement experimental results, MD studies were performed to determine the positioning of *O*-acetyl groups on model COPS. Computational modeling revealed the importance of this acetylation and its solvent flexibility.

### 4.2. Directed Evolution of Glycosyltransferases

Directed evolution is a method that has been used to engineer carbohydrate-producing enzymes with altered substrate specificity. In this method, iterative rounds of mutations are performed and through library assay screening positive hits can be identified. Recent work has leveraged a FACS-based assay during the directed evolution of an α1→4 fucosyltransferase with higher natural activity and the ability to use fluorescent acceptors [61]. Crystal structure elucidation of the mutant enzyme revealed structural elements that led to this functional activity. Although not completely widespread in the field, directed evolution has been applied to many glycosyltransferases [62,63,64,65,66,67]. Recent description of ways that machine learning can coupled to directed evolution can help in screening protein variants more expeditiously [68].

## 5. Conclusions

Glycans located on the cell surface of pathogenic bacteria serve as valuable components of life-saving vaccines. While currently licensed glycoconjugate vaccines contain heterogenous glycans, comparing these to bacterial vaccines using homogeneous glycans will help elucidate the mechanisms of protection for various pathogens. As researchers continue to explore ways to synthesize homogenous glycans on a large scale, efforts are being made to improve enzymatic synthesis as discussed in this review. The most well characterized methods for chemoenzymatic synthesis of meningococcal oligosaccharides were discussed. While *N. meningitidis* is not a pathogen that is at risk for antibiotic resistance, the lessons learned from their chemoenzymatic synthesis could inform future vaccine development against pathogens prioritized for antibiotic resistance. This review has discussed some advances in chemoenzymatic synthesis of oligosaccharides for vaccines against selected WHO-prioritized bacteria. Finally, homogenous vaccines of the future will most likely continue to use multidisciplinary approaches to leverage wet-laboratory experimental data and molecular modeling and dynamics to improve glycoconjugate formation. More research needs to be carried out to advance the delineation of the relationship between vaccine structure, immunogenicity and protection.

## Figures and Tables

**Table 1:** Differences of chemoenzymatic and chemical synthesis.

Chemoenzymatic Synthesis	Chemical Synthesis
Efficient synthesis of complex oligosaccharides under milder conditions. Specificity of enzymes allows for fewer reaction steps in a more streamlined process.	More complex and inefficient synthesis process. Requires a large amount of protecting group manipulations.
No carbohydrate-protecting groups needed because of regiospecificity.	Glycosylation reaction requires a lot of time and can be laborious.

**Table 2:** Types of bacteria that are antibacterial resistant (from WHO).

WHO Priority Level	Bacteria	Antibiotic Resistance
Highest	*Mycobacterium tuberculosis*	Fluoroquinolone
		Rifampicin
		Isoniazid
Critical	*Acinetobacter baumannii* *Pseudomonas aeruginosa* *Enterobacteriaceae* spp.	Carbapenem
High	*Enterococcus faecium*	Vancomycin
	*Staphylococcus aureus*	Methicillin, Vancomycin
	*Helicobacter pylori*	Clarithromycin
	*Campylobacter* spp.	Fluoroquinolone
	*Salmonellae*	Fluoroquinolone
	*Neisseria gonorrhoeae*	Cephalosporin, Fluoroquinolone
Medium	*Streptococcus pneumoniae*	Penicillin
	*Haemophilus influenzae*	Ampicillin
	*Shigella* spp.	Fluoroquinolone
